# The Low-Density Lipoprotein Receptor-Related Protein-1 Is Essential for Dengue Virus Infection

**DOI:** 10.3390/v16111692

**Published:** 2024-10-30

**Authors:** Vivian Huerta, Alejandro M. Martin, Mónica Sarría, Osmany Guirola, Alexis Yero, Yassel Ramos, Dianne Pupo, Dayron Martin, Tea Carletti, Luis G. González-Lodeiro, Alessandro Marcello, Glay Chinea

**Affiliations:** 1 Department of System Biology, Direction of Biomedical Research, Center for Genetic Engineering and Biotechnology, Havana 10600, Cuba; amiguelmd67@gmail.com (A.M.M.); monica.sarria@nauta.cu (M.S.); osmany.guirola@cigb.edu.cu (O.G.); ayerodiaz@mgh.harvard.edu (A.Y.); yassel.ramos@cigb.edu.cu (Y.R.); dianne.pupo.gomez@usherbrooke.ca (D.P.); dayron.martin@ihatuey.cu (D.M.); luis.gonzalez@cigb.edu.cu (L.G.G.-L.); glay.chinea@cigb.edu.cu (G.C.); 2 Laboratory of Molecular Virology, International Centre for Genetic Engineering and Biotechnology, 34149 Trieste, Italy; tea.carletti@icgeb.org (T.C.); marcello@icgeb.org (A.M.)

**Keywords:** domain III, cellular receptor, virus–receptor interaction

## Abstract

Dengue virus (DENV) causes the most prevalent and rapidly spreading arboviral disease of humans. It enters human cells by receptor-mediated endocytosis. Numerous cell-surface proteins were proposed as DENV entry factors. Among these, the phosphatidylserine receptor TIM-1 is the only one known to mediate virus internalization. However, several cellular models lacking TIM-1 are permissive to DENV infection, suggesting that other receptors exist. Here, we show that the low-density lipoprotein receptor-related protein-1 (LRP1) binds DENV virions by interacting with the DIII of the viral envelope glycoprotein. DENV infection is effectively inhibited by the purified receptor at 5 × 10^−8^ mol/L, and the interaction of the envelope protein with LRP1 is also blocked by a natural ligand of LRP1. The depletion of LRP1 causes 100-fold lower production of infectious virus than controls. Our results indicate that LRP1 is another DENV receptor, thus becoming an attractive target to evaluate for the development of effective antiviral drugs against DENV.

## 1. Introduction

The Dengue complex consists of four viral serotypes (DENV1–4) of a mosquito-borne virus of the family *Flaviviridae*; genus Flavivirus. DENV-caused disease encompasses a wide spectrum of clinical manifestations ranging from a mild self-limited disease known as Dengue Fever to the life-threatening manifestations of Dengue Hemorrhagic Fever [1]. The global incidence of DENV infections has grown dramatically in recent decades, making this virus a worldwide public health concern [2].

DENV particles consist of a nucleoprotein core formed by RNA and the capsid protein. This core is surrounded by a host-derived lipid bilayer with two glycoproteins inserted: the membrane (M) protein or its precursor (prM), found in immature virions, and the envelope (E) protein [3]. The latter is organized in three structural domains: DI, DII and DIII [4]. Of these, the DIII has been identified as directly involved on virus–receptor interactions [5,6,7,8]. However, no specific receptor has been identified that binds the virus through this region of the E protein.

DENV enters the cell by receptor-mediated endocytosis [9]. Early steps of DENV infection comprise interactions with soluble and membrane-bound attachment factors, signaling receptors and internalization receptors [10]. There are two main scenarios for these events: the primary infection with any of the four virus serotypes and a secondary heterologous infection [10]. In the latter, antibodies generated during the first infection cross-reacting but not cross-neutralizing the infecting serotype act as soluble attachment factors that bridge the virus to Fcγ receptors in the cell surface. Interaction with Fcγ receptors also appears to facilitate virus internalization [11,12] and to generate intracellular signaling that suppresses antiviral responses [13,14]. All these factors together lead to a significant increase in the number of infected cells and virus production. This phenomenon, known as antibody-dependent enhancement, results in an increased susceptibility of infected individuals to the development of severe forms of DENV-caused disease.

DENV uses interactions with soluble proteins [15,16] and attachment factors at the plasma membrane-like cell surface, such as heparan sulfate glycosaminoglycans [16], and pathogen-binding C-type lectins on cells of the innate immune system [17,18,19,20,21] to increase the efficiency of the binding to the cell surface. After attachment, virus entry take place via receptor-mediated endocytosis, which ultimately leads to the delivery of the viral genome into the cytoplasm [19]. So far, only the T-cell immunoglobulin and mucin domain 1 (TIM-1) receptor, which binds phosphatidylserine moieties on the surface of the viral membrane, has been convincingly shown to mediate DENV endocytosis [20]. However, some cellular models that do not express TIM-1 are still sensitive, at low levels, to DENV infection. Therefore, we have not yet reached a complete understanding of the process by which DENV enters mammalian and mosquito cells. Other molecules capable of binding protein E may possibly participate in receptor-mediated endocytosis of this pathogen.

The regulation of lipid metabolism significantly influences the efficiency of DENV infection and replication in vitro and in vivo [21,22,23,24]. Sequence stretches on DENV E and capsid proteins resemble receptor-binding motifs of ApoE [25]. ApoE is present in many lipoproteins and interacts with several receptors of the low-density lipoprotein receptor (LDLR) family [26]. Bovine lactoferrin partially inhibits DENV and Japanese encephalitis infection, presumably competing for binding to the LDLR [27,28]. Collectively, these results suggest that the LDLR may be involved on DENV entry to target cells. Yet, conclusive evidences of the participation of the LDLR on DENV attachment and/or entry have not been obtained.

The LDLR family comprises at least 13 separate cell receptors that mediate lipoprotein internalization, although some also play a role in the regulation of cellular physiology and intracellular signaling [29]. In the context of early events of the DENV infectious cycle, we decided to focus our interest on a different member of the family, the LRP1 receptor. This protein plays a role not only in lipoprotein endocytosis but also in the internalization of protease–serpin complexes and, particularly, of activated alpha-2-macroglobulin (α2M*):protease complexes [30]. We found that proteins participating in these processes are significantly enriched within the plasma DENV interactome [31,32]. Also, we showed that α2M*, a ligand of LRP1, directly binds DENV virions and favors DENV infection [33]. In this work, we evaluated LRP1 as a putative DENV receptor. In particular, we investigated the effect of receptor silencing in human hepatoma cells (Huh-7) and the virus–receptor interaction.

## 2. Materials and Methods

### 2.1. Cells and Viruses

Human hepatoma-derived cells (Huh7) and human embryonic kidney cells (HEK-293T) cells were grown under standard conditions at 37 °C, 5% CO_2_ in Dulbecco’s modified Eagle’s medium (DMEM) + GlutaMAX supplemented with 10% of fetal bovine serum (FBS), adding heat-inactivated FBS to a final concentration of 10% (*v*/*v*).

### 2.2. Proteins

Recombinant proteins DVE1 (code MBS144575), DVE3 (code MBS 144886) and DVE4 (code MBS 145224) were purchased from MyBioSource, Inc., San Diego, CA, USA. The procedures for the expression, purification and general characterization of the proteins corresponding to domain III of the E protein of the four DENV serotypes, namely, DIIIE1–4, were conducted as previously described [33]. Receptor-activated α2M was purified from human plasma also following the procedure described elsewhere [33].

The human protein associated with the LRP1 receptor, known as RAP, was obtained by expressing in *E. coli* a gene fragment coding for this molecule. The gene for the RAP protein was amplified from the cDNA by PCR using the oligonucleotides CATATGTACTCGCGGGAGAAGAACCAG and CTCGAGTCAGAGTTCGTTGTGC, bearing on their 5′ end the recognition sequence for the restriction enzymes Nde I and Xho I (in boldface in the sequence). The amplified fragment was used to construct pET-RAP, a plasmid that codes for the intracellular synthesis of RAP under control of the T7 promoter. For protein expression, a pET-RAP plasmid was transformed into the *E. coli* strain BL21. The recombinant RAP protein was obtained with a tag of six consecutive histidines at the N-terminus. The RAP protein was purified from the supernatant by immobilized metal affinity chromatography using Ni-NTA agarose and was eluted with a linear gradient of 10 to 300 mM imidazole in phosphate-buffered saline (PBS, 10 mM of Na_2_HPO_4_, 1.8 mM of KH_2_PO_4_, 2.7 mM of KCl, 137 mM of NaCL, pH 7.4).

Recombinant proteins sLRP1_CII, sLRP1_CIII and sLRP_CIV were purchased from R&D Systems, Minneapolis, MN, USA. The Flavivirus-reactive mAb 4G2 was provided by the Unit for Production of Monoclonal Antibodies at the Center for Genetic Engineering and Biotechnology in Sancti Spiritus, Cuba. Mab MCA1965, was purchased from AbD serotec, Hercules, CA, USA, and Mab 5A6 was donated by Dr. D.K. Strickland.

### 2.3. Affinity Purification of LRP1

Frozen human plasma was obtained from local blood banks and kept at −80 °C until used. One hundred twenty-five milliliters of pooled human plasma from healthy donors were dialyzed against 50 mM of HEPES at pH 6, 60 mM of NaCl, 1 mM of EDTA, centrifuged at 10,000× *g* for 30 min at 4 °C and filtered through a 0.45 µm membrane. The resulting sample was fractionated using anion exchange chromatography [34,35]. To this end, conditioned plasma was loaded onto a chromatography column packed with 10 mL of DE-52 gel previously equilibrated with the same buffer used for the dialysis. The sample was eluted by step-washing of the column with 50 mM of HEPES at pH 6, 1 mM of EDTA and an increasing concentration of NaCL (0.3 M, 6 M and 1 M). The elution of LRP1 was detected by a ligand-binding assay in a dot blot format. In the assay, the nitrocellulose membrane was sensitized with the elution fractions from DE-52 chromatography, followed by an incubation with biotinylated alfa 2-macroglobulin and a streptavidin peroxidase conjugate to detect the bound ligand. Positive fractions to LRP1 detection were pooled and dialyzed against 50 mM of HEPES at pH 7.0, 120 mM of NaCl, 5 mM of CaCl_2_, 1 mM of MgCl_2_ and supplemented with a protease inhibitor cocktail (1 µg/mL of leupeptin, 1 µg/mL of pepstatin A, 1 µg/mL of a soybean trypsin inhibitor and 1 mM of PMSF) for loading in the affinity chromatography using receptor-activated α2M as an immobilized ligand. Then, the conditioned sample was loaded into a column packed with 1 mL of the affinity matrix at a flow of 10 cm/h. The column was washed with 100 column volumes of the loading buffer. The bound protein was eluted with 50 mM of HEPES at pH 6.0, 1.2 M of NaCl and 25 mM of EDTA. The eluted protein sample was dialyzed against the loading buffer, filtered through 0.2 µm and stored at −20 °C until used.

### 2.4. Surface Plasmon Resonance Analysis (SPR)

SPR analyses were performed on a Biacore X unit (Cytiva, Marlborough, MA, USA). Proteins were covalently immobilized on CM5 chips using amine-coupling chemistry. On each chip, flow channel (FC) 1 was used to immobilize the specific protein ligand, and FC2 was prepared for background control. The surface of the FC1 channel was activated for 7 min with a 1:1 mixture of 0.1 M N-hydroxysuccinimide and 0.1 M N-ethyl-N9-(3-diethylamino-propyl) carbodiimide. DIIIE1 (30 µg/mL) and RAP (20 µg/mL) proteins were dissolved in a 10 mM sodium acetate buffer at pH 5 and loaded at 5 µL/min into the activated surface. 

Immobilization of sLRP1_CIV was carried out at a concentration of 10 µg/mL in a 10 mM sodium acetate buffer at pH 4.0. All remaining free activated groups were blocked by the application for 7 min of 1 M of ethanolamine, pH 8. The FC2 channel received only the activating and blocking applications. All SPR experiments were performed at a flow rate of 25 µL/min using HEPES 10 mM, pH 7.4, 0.15 M of NaCl, 3 mM of CaCl_2_, 0.005% surfactant P20 (HBS-Ca) as a running buffer. The interacting surface was regenerated with a pulse of 5 µL of 10 mM NaOH. Results were analyzed using the BIAevaluation ver. 4.1 software application package (General Electric, Boston, MA, USA).

### 2.5. Pull Down Assays

Purified DIIIE1–4 and RAP were immobilized at a ligand density of 4 mg/mL in CNBr-activated Sepharose (Cytiva, Marlborough, MA, USA), as recommended by the provider. A background control matrix was prepared following the identical procedure but without any immobilized protein.

To obtain the microsomal fraction from Huh7 cells, subconfluent monolayers were scraped from the flasks, washed three times with PBS, resupended in 1 mL of hypotonic buffer (20 mM of Tris pH 7.4, 10 mM of KCl, 5 mM of EDTA, 1 mM of PMSF, 1 µg/mL of peptstatin, 1 mM of leupeptin) and incubated 20 min on ice. All subsequent steps were performed at 4 °C. Sucrose was added to a final concentration of 250 mM, the cell suspension was transferred to a Dounce homogenizer and disrupted by 3 strokes followed by brief sonication. Cell homogenates were first centrifuged at 1,000× *g* to remove nuclei, and supernatants were then centrifuged at 10,000× *g* for 20 min to pellet the mitochondrial fraction. The microsomal fraction was obtained by centrifuging post-mitochondrial supernatant at 100,000× *g* for 1 h. The resulting 100,000 g pellet was resuspended at a concentration of 10 mg/mL in HBS-Ca and stored at −80 °C until use.

The microsomal fraction was split in 6 identical aliquots, each of which was incubated with 1 mL of the affinity matrices and background control gel for 18 h at 4 °C in a rotary mixer. The affinity gels were washed with 100 matrix volumes of HBS-Ca, 0.005% of n-Octyl-β-D-Glucopyranoside. Bound proteins were eluted by incubating the gels twice for 15 min at 25 °C with 2 mL of 0.1 M acetic acid followed by 1 mL of 3 M urea, 1 mM of EDTA. The three eluates from each gel were then pooled, concentrated and adjusted to pH 8.0 by adding 0.2 mL of Tris-HCl 2 M, pH 8.0. Protein digestion, Selected Reaction Monitoring analysis and data processing were performed as described [31].

### 2.6. Virus Assays

Both viruses DENV2 (strain S16803, NIBSC code S16803) and Vesicular Stomatitis Virus (VSV strain Indiana) were propagated and titrated by plaque formation assays performed in Vero cells. Plaque formation assays were performed in 24-well plates with cell monolayers at 90% confluence. Serial dilutions of virus inoculum were diluted in DMEM 2% FBS, added to cells and incubated for 2 h in the CO_2_ incubator. Next, viral dilutions were removed, cell monolayers were washed twice with DMEM 2% FBS and incubated for 5 days at 37 °C in a high-density medium (MEM supplemented with non-essential amino acids, 1% FBS, 1% carboxymethylcellulose) in order to propitiate the appearance of lytic plaques. The plaques were visualized by staining with 0.1% Naphtol Blue Black in 0.15 M of sodium acetate. Two replicates were used per experimental point in each assay.

For the purification of DENV2, monolayers of Vero cells were infected at a moi 0.01, washed with fresh DMEM without FBS and maintained in a serum-free medium for 96 h. Cell supernatants were clarified by centrifugation at 5000 rpm for 30 min. Viruses were sedimented by ultracentrifugation at 30% sucrose in a 5 mM HEPES solution, 150 mM of NaCl, 0.1 mM of EDTA at 32,000 rpm for 9 h. Virus pellets were resuspended in the same buffer without sucrose. The purified virus preparation was analyzed by SDS-PAGE followed by Coomasie blue staining. Visible protein bands corresponded to E and pre-M proteins as confirmed by Western blotting using specific Mabs for each protein.

Inhibition of infection with chromatography fractions was performed using a plaque formation assay in Huh7 cells. Cells were seeded at 10^4^ per well in 24-well plates and kept for 18 h at 37 °C in a CO_2_ incubator. The DENV2 inoculum was diluted in DMEM without FBS and adjusted to obtain 50 PFU/well. Chromatography fractions were diluted in DMEM without FBS, added to the virus inoculum to obtain a final protein concentration of 10 µg/mL and incubated for 30 min at 37 °C. For virus control, only PBS was added to the virus inoculum previous to the incubation. Next, virus–sample mixtures were added to cell monolayers, and infection proceeded for 2 h at 37 °C. Afterwards, cell monolayers were washed, and a high-density medium was added. Viral plaques were visualized 5 days post-infection.

For the evaluation of virus yield after transfection with shRNAs, Vero cells were seeded in 96-well plates and cultured until monolayers reached 90% confluence. Viruses were diluted in DMEM, 2% FBS for an m.o.i of 0.1 in case of DENV2 and 0.35 for VSV. Monolayers were washed once with DMEM 2% FBS, and, subsequently, 100 µL of virus inoculum were added. After 1 h of incubation, viral inoculum was removed and replaced by 100 µL of fresh DMEM 2% FBS. To evaluate viral production, a 24 h supernatant was collected and assayed for infectious virus by plaque assay on Vero cells.

### 2.7. Gene Silencing

Target sequence within the hLRP1 mRNA (5′-GATCCGTGTGAACCGCTTTAA-3′) (NM_002332.2, RNAi Consortium at http://www.broadinstitute.org/rnai/public, accessed on 17 November 2017) was confirmed to match within a CDS on the LRP1 gene sequence and to be unique for this gene. Template sequences encoding a short hairpin RNA were generated and cloned into a de pKLO.1-TRC shRNA lentivector:

Forward oligo:

5′-CCGGGATCCGTGTGAACCGCTTTAACTGCAGTTAAAGCGGTTCACACGGATCTTTTTG-3′

Reverse oligo

5′-AATTCAAAAAGATCCGTGTGAACCGCTTTAACTGCAGTTAAAGCGGTTCACACGGATC-3′

Oligonucleotides (Integrated DNA Technologies, Coralville, IA, USA) were annealed, phosphorylated and ligated into AgeI and EcoRI sites of the pKLO.1 vector. Plasmid-containing clones were selected by resistance to ampicillin and checked by restriction analysis and sequencing.

Monolayers of 293T cells were co-transfected with packaging construct (psPAX2) coding for gag, pol, tat and rev of HIV: envelope plasmid (VSV-G) and pLKO.1 based shRNA-LRP1. As negative controls were used, two pLKO.1 constructs were designed to activate the silencing machinery but without targeting any gene in the cells: shRNA-scramble. Transfection was performed in 10 cm plates by the Ca-phosphate method using 10 µg of DNA mixed in the ratio pLKO.1_shRNA:psPAX:VSV-G 4:3:1. After 24 h, media-containing transfection mixtures was replaced by fresh DMEM, 10% FBS. The lentivector-containing supernatants were harvested at 48 and 72 h post-transfection. Collected supernatants were clarified by centrifugation followed by filtration through 0.45 µm, aliquoted and store at −80 °C until used.

Lentivirus-containing supernatants were used at indicated dilutions to infect Huh7 cells. Infection was performed in DMEM, 10% FBS, 5 µg/mL of polybrene. After 18 h, the lentivirus inoculum was removed and replaced by selection medium: DMEM, 10% FBS, 1 µg/mL of Puromycin (InvivoGen, San Diego, CA, USA). Efficiency of transduction was estimated by the detection of GFP expression using FACS.

Huh7 cells transduced with either the LRP1-specific shRNA or a scrambled shRNA control were infected at day 7 post-transduction with DENV2, and infectious particle release was quantified by titration 24 h post-infection.

### 2.8. Denaturing Polyacrilamide Gel Electrophoresis (SDS-PAGE)

One microgram of protein of chromatography fractions was diluted in a non-reducing sample buffer, loaded in a 6–15% gradient polyacrylamide denaturing gel and separated under standard conditions [36]. Proteins were detected using silver staining [37]. For the evaluation of LRP1 expression after gene silencing, 10^5^ cells were lysed in a non-reducing sample buffer, separated in an 8% polyacrylamide gel and subjected to Western blotting analysis.

### 2.9. Western Blotting

Separated proteins were transferred to nitrocellulose membranes under standard conditions [38]. Afterward, transfer membranes were incubated for 1 h at 25 °C with HEPES 20 mM at pH 7.2, 0.05% Tween 20, 1 mM of CaCl_2_. Membranes were then blocked for 1 h using 1% Bovine Serum Albumin (BSA) and incubated with a 10 µg/mL dilution of the primary antibody for 16 h at 4 °C. Next, blots were washed three times with PBS-T, and antibody binding was detected using an anti-mouse antibody conjugated to HRP. For visualization, an ECL development solution was used in the evaluation of LRP1 expression after gene silencing and 3,3′-diaminobenzidine (Sigma-Aldrich, Burlington, MA, USA) in the analysis of affinity chromatography fractions.

### 2.10. ELISA

Ninety-six well microtiter plates were coated for 1 h at 37 °C with 50 µL per well of the corresponding protein at 10 µg/mL or 2 × 10^6^ pfu of purified DENV2 in carbonate buffer at pH 9.6. The plates were blocked with 2% (*w*/*v*) BSA in PBS, 0.05% Tween 20 (PBS-T) for 1 h at 37 °C. Recombinant sLRP-CI-IV were diluted in HBS-Ca, added to coated wells and incubated for 1 h at 37 °C. After three washes with the binding buffer, bound proteins were detected using an anti-human Ig, Fc specific-HRP conjugate. The development was carried out with 1 mg/mL o-phenylenediamine dihydrochloride, 0.1% hydrogen peroxide in a 0.05 M phosphate-citrate buffer, pH 5.0. The developing reaction was stopped with 3 M of H_2_SO_4_, and results were read at 492 nm in a microplate reader.

### 2.11. Statistical Analysis

GraphPad Prism V5.3 was used to perform an unpaired Student’s *t*-test to calculate *p* values [39]. Differences with *p*-values of < 0.05 were considered significant (* *p* < 0.05, ** *p* < 0.01, *** *p* < 0.001).

## 3. Results

### 3.1. LRP1 Depletion Inhibits DENV2 Infection

**Figure 1:**
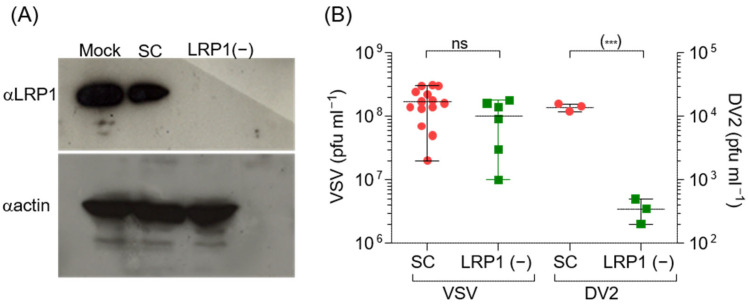
LRP1 is essential for DENV2 infection. (**A**) Western blotting analysis of the silencing of LRP1 expression on cells transfected with scramble shRNA (SC, red circles) or with LRP1-shRNA (green squares). LRP1 was detected using the Mab 5A6 that recognizes the 85 kDa β chain of the receptor [44]. Detection of βactin was performed on the same membrane. (**B**) Effect of the transduction of LRP1-shRNA on DENV2 infection. Transduced cells were infected at day 7 with DENV2 (moi of 0.1) or vesicular stomatitis virus (VSV, moi 0.35). Virus yield was measured on 24 h post-infection supernatants. GraphPad Prism V5.3. was used for the unpaired Student’s *t*-test, where *** *p* < 0.001.

To investigate whether the presence of LRP1 on the cell surface could impact the efficiency of infection, we examined the effect of shRNA-mediated LRP1 knock-down on viral yield. The assay was performed in Huh7 hepatoma cells, which are extensively used in *Flaviviridae* research and physiologically relevant for DENV infection [40], and they express LRP1 [41]. Huh7 cells can be efficiently transduced by lentiviral vectors without alterations to key hallmarks of the hepatic phenotype [42]. Western blotting analysis revealed that LRP1 expression was efficiently inhibited by day 6 post-transduction (Figure 1A). Viral yield in the cells transduced with the LRP1-specific shRNA was 100-fold lower than in the cells transduced with the scrambled control (Figure 1B). These results show that LRP1 plays a central role on the DENV replication cycle. Meanwhile, VSV, which presumably can use LRP1 as an alternative to LDLR [43], while significantly less efficient than the latter, exhibits only a slight decrease in virus titer.

### 3.2. Ligand-Purified LRP1 Inhibits DENV2 Infection in Hepatocytes

**Figure 2:**
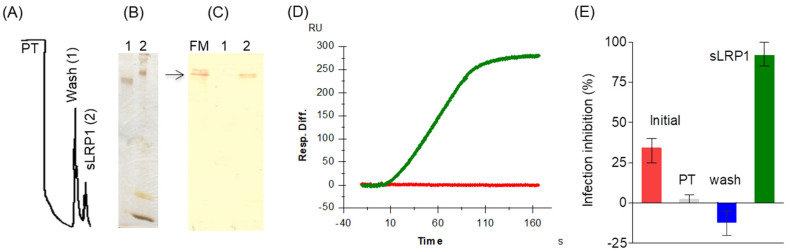
Purified sLRP1 inhibits DENV2 infection. (**A**) Chromatographic profile of the affinity chromatography using receptor-activated α2M as immobilized ligand. PT: pass-through. Numbers in parenthesis identify the lane in SDS-PAGE (**B**) and Western blotting analysis (**C**). In (**C**), membrane was probed with an anti-LRP1 Mab (MCA1965) that recognizes the alpha chain of the receptor. Microsomal fraction (FM) of Huh7 cells was used as control of migration of α chain of LRP1. (**D**) Binding analysis of purified sLRP1 to recombinant RAP protein using SPR. RAP protein was immobilized on the surface of the chip, and purified sLRP1 (green line) was injected and diluted in HBS-Ca at 1 µg/mL. Red line corresponds to the application of the initial sample of the affinity chromatography at the same protein concentration. Resp. Diff denotes the response obtained by automatically subtracting the signal of the reference channel from that of the specific channel. (**E**) Inhibition of DENV2 infection in Huh7 cells after incubation with chromatography fractions. Results from one representative experiment out of three independent experiments. Error bars represent the range of three sample replicates.

One testable prediction of the hypothesis that LRP1 participates in early steps of virus–cell interaction would be to inhibit DENV infection by competition with a soluble form of LRP1. Therefore, we purified the LRP1 ectodomain (sLRP1) from human plasma by using receptor-activated α2M as an affinity ligand (Figure 2A–C), taking advantage of the fact that, under physiological conditions, sLRP1 is shed from cell surfaces into body fluids in a soluble, active ligand-binding form [45]. SPR experiments with the purified sLRP1 on chips containing immobilized RAP, which is the intracellular receptor chaperone of LRP1 [30], confirmed that it retained its normal ligand-binding activity (Figure 2D).

An inhibition-of-infection assay was then set up in Huh7 cells where a DENV2 preparation was pre-incubated either with purified sLRP1 or with different fractions collected during the purification process (the initial sample, the pass-through and the washing eluate). As previously reported, on these experimental conditions, the human plasma fraction used as a starting sample shows partial inhibitory activity against the DENV2 infection of Huh7 hepatocytes [34]. Neither the pass-through nor the washing eluate exhibited inhibitory activity, while the eluate containing sLRP1 at 5 × 10^−8^ mol/L exhibited an inhibitory activity close to 90%, indicating a significant enrichment of inhibitory activity (Figure 2E). These results indicate a direct interaction of LRP1 with DENV2 and are consistent with the involvement of LRP1 in the initial stages of the DENV life cycle. The high potency of the observed inhibitory effect suggests that it involves the blockage of critical functional sites on the viral surface (i.e., direct competition with cell-bound LRP1) rather than the indirect occlusion of binding sites for other cell receptors.

### 3.3. LRP1 Interacts with DENV Particles

**Figure 3:**
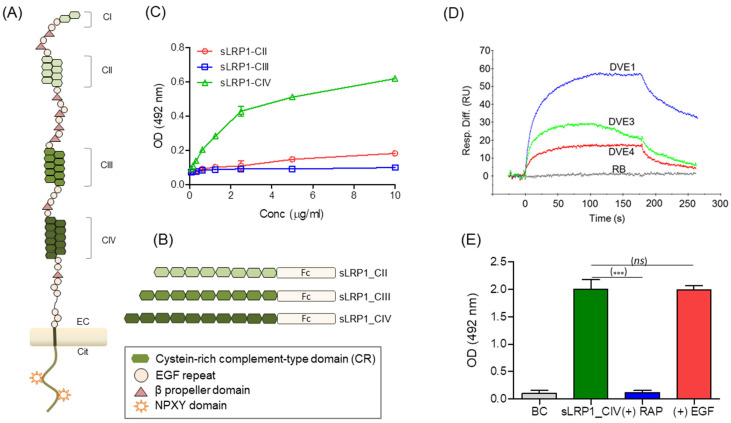
Interaction of DENV2 with ligand-binding clusters of LRP1. Schematic representation of (**A**) LRP1 and (**B**) the recombinant proteins sLRP1-CII, sLRP1-CIII and sLRP1-CIV. (**C**) Evaluation of the interaction of recombinant proteins sLRP1-CII, sLRP-CIII and sLRP1-CIV with purified DENV2. (**D**) Interaction of sLRP1-CIV with recombinant preparations of the ectodomain of the E protein of DENV serotypes 1, 3 and 4 (DENVE1, DENVE3 and DENVE4) by SPR. sLRP1-CIV was immobilized in the surface of the chip and recombinant DENV E proteins were injected and diluted in HBS-Ca at 10 µg/mL. Resp Diff: signal obtained by online subtraction of background control channel. (**E**) Binding competition by ELISA. Plates were coated with DENVE1 protein, and the binding of sLRP1-CIV at 5 µg/mL was evaluated after the incubation for 30 min at 37 °C with EGF or the LRP1 ligand protein RAP, both proteins used at 5 µg/mL. BC: background control. Results are representative of two independent analyses. Error bars represent the range of three replicates. GraphPad Prism V5.3. was used for the unpaired Student’s *t*-test, where *** *p* < 0.001.

LRP1 is a heterodimeric type I integral membrane protein comprising an extracellular 515 kDa α chain and a non-covalently linked cytoplasmic and transmembrane 85 kDa β chain (Figure 3A). The interaction of LRP1 with extracellular ligands typically takes place through the complement-type repeats that are individually known as ligand binding domains (LBDs) and are presented in four clusters of 2, 8, 10 and 11 LBDs on the α chain denominated CI, CII, CIII and CIV, respectively. Of these, clusters CII–CIV are those that bind most of the LRP1 ligands [30].

In order to investigate whether DENV particles can bind LRP1, we analyzed the interaction of a purified preparation of DENV2 adsorbed to ELISA plates with three chimeric proteins denoted sLRP1_CII, sLRP1_CIII and sLRP1_CIV, containing clusters II, III or IV from LRP1 fused to a human IgG1 Fc domain (Figure 3B). As shown in Figure 3C, DENV2 bound efficiently to the fusion protein containing cluster IV, but not those containing clusters II or III. Considering that the construct of sLRP1-CIV protein comprises only the 11 LBDs of CIV (Figure 3B), these results directly imply that, similar to the interaction with other extracellular ligands of LRP1, the complement-type repeats (Figure 3A) are involved in the interaction with DENV2.

### 3.4. The E Protein of DENV Binds LRP1

The E protein covers most of the solvent-accessible surface of DENV virions [46]. Therefore, we next investigated if protein E mediates the interaction between DENV and LRP1 and whether this interaction is conserved for the other viral serotypes. To this end, we examined by SPR the binding of recombinant proteins encompassing the E ectodomain from DENV serotypes 1, 3 and 4 (DVE1, DVE 3 and DVE 4 in Figure 3D) to sLRP1_CIV immobilized on an SPR chip. All three recombinant E proteins bound the sLRP1_CIV-derivatized surface obtaining an almost three times higher signal for DVE1 followed by DVE3 and DVE4 (Figure 3D). This result demonstrates that the DENV-LRP1 cluster IV interaction is mediated by protein E and that it is preserved in serotypes other than DENV2, although with probable differences in reactivity among the four viral serotypes. DVE1 was then selected to test whether the RAP protein was able to block the interaction using an ELISA format. Results showed that, similar to many other LRP1 ligands, the pre-incubation of sLRP1-CIV with RAP abolished the interaction with DVE1, while no effect was observed when another protein non-relevant for this system i.e., the human Epidermal Growth Factor (EGF), was used as a competitor (Figure 3E).

### 3.5. The Interaction of E with LRP1 Is Mediated by DIII of Protein E

**Figure 4:**
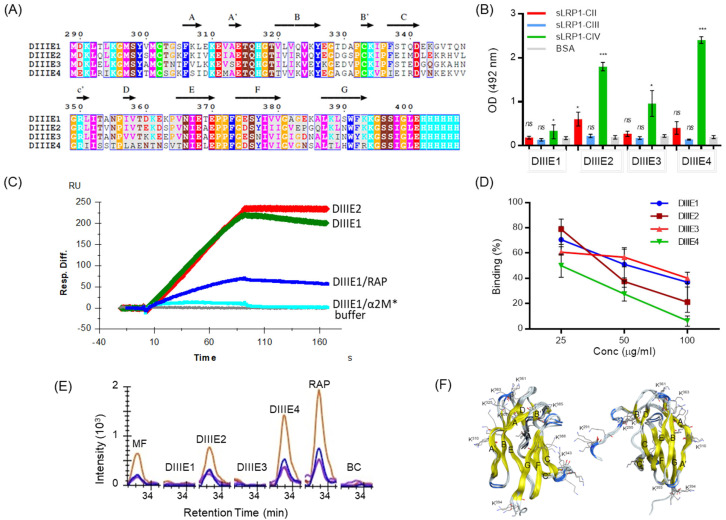
Interaction of recombinant DENV E DIII proteins with LRP1. (**A**) Multiple sequence alignment of DIIIE1–4 proteins (residue numbers according to DENV1, 2 and 4). The alignment was performed using the ClustalW application [49]. The arrows denote β-strands. Residues are colored according to the physico-chemical properties and conservation according to ESPript program [50]. (**B**) Interaction of DIIIE1–4 proteins with sLRP1-CII, sLRP1-CIII or sLRP1-CIV by ELISA. Results are mean and SEM of three independent experiments. An unpaired Student’s t-test was used to compare the response against sLRP protein against BSA control, where * *p* < 0.05, and *** *p* < 0.001. (**C**) Binding analysis of sLRP1 to immobilized DIIIE1 by SPR. sLRP1 at 1 µg/mL was loaded over the DIIIE2 and DIIIE1 surfaces. For this later analysis, sLRP1 injection was performed without or after pre-incubation with RAP at (DIIIE1/RAP), or receptor-activated α2M (DIIIE1/α2M*). Resp. Diff.: non-specific signal was subtracted online using a reference channel without any immobilized protein. (**D**) Blocking of sLRP1-DENV2 binding. A concentration of sLRP1-CIV at 5 µg/mL was incubated with a concentration of 10 µg/mL of DIIIE1–4 for 1 h before the addition to DENV2-coated plates. Bound sLRP1-CIV was detected using an anti-human Fc Ig-POD conjugate. (**E**) Binding to LRP1 present in the microsomal fraction of Huh7 cells. Pull down assay using recombinant DIIIE1–4 proteins as bait and detection by Mass spectrometry using Selected Reaction Monitoring. MF: microsomal fraction. BC: background control. Data show transition signals of the surrogate peptide _365_IVFPHGITLDLVSR_378_ (Swissprot Accession number Q07954). Samples were analyzed in duplicate. Similar results were obtained for other five proteotypic peptides (Supplementary Table S1). (**F**) Cartoon representation of the 3D structure of DIII. In yellow, the β-strands and lysine residues are represented, shown in wire-frame representation. Residue numbers correspond to DENV1, 2 and 4.

A number of results indicate that DIII of E protein of flaviviruses participate in the interaction with cell surface molecules that play key roles in virus binding [5,6,8,47,48]. We thus decided to investigate whether the protein E-LRP1 interaction is mediated by this region of E protein. For this purpose, we used recombinant DIII proteins of the four viral serotypes, denoted DIIIE1, DIIIE2, DIIIE3 and DIIIE4 (residues 289–400 of DENV1, 2, 4 and 287–388 of DENV3) (Figure 4A), to coat ELISA plates, which were probed with the sLRP1-derived chimeras (Figure 3B). DIIIE from all four serotypes bound sLRP1-CIV (Figure 4B). DIIIE2 also bound sLRP1_CII weakly, although this reactivity was undetectable for the other three serotypes, and no binding to sLRP1_CIII was detected for any of the recombinant DIII preparations. In this assay format, the recombinant protein corresponding to DIII of DENV1 (DIIIE1) was the one showing the lowest reactivity. This apparent contradiction with the results obtained by SPR with DENVE1, 3 and 4 (Figure 3D) may reflect a higher susceptibility of the recombinant DIIIE1 protein to lose reactivity when adsorbed to the surface of the plate. In fact, DIIIE1 showed similar reactivity with purified sLRP1 as DIIIE2 when compared in SPR (Figure 4C). Specificity of the binding to the DIIIE1 surface was evidenced using a competition assay with two LRP1 ligands, RAP and receptor-activated α2M. As shown in Figure 4C, binding to DIIIE1 surface decreases significantly when RAP and receptor-activated α2M were incubated with sLRP1 before the analysis.

The ability of recombinant DIII proteins to block the binding of sLRP1-CIV to DENV2 (Figure 4D) was also evaluated. In the assay, DIIIE1–4 proteins were incubated in solution with sLRP1-CIV before its addition to DENV2 particles adsorbed in ELISA plate wells. The results show that recombinant DIII proteins of the different viral serotypes are able to block sLRP1-CIV to DENV2 to different levels with more than 95% of inhibition exhibited by DIIIE4 at the maximal protein concentration evaluated in the assay (100 µg/mL) followed by DIIIE2 > DIIIE1 > DIIIE3. This result shows that DIII is a dominant site of interaction, although it does not rule out the existence of other regions within the E protein that may contribute to the binding to sLRP1-CIV. This result also highlights the fact that DIII surface patches involved in the interaction with LRP1 are sufficiently similar that a recombinant DIII from one serotype can effectively compete for LRP1 binding to the virus particle of a different serotype.

The binding of recombinant DIII to full-length LRP1 was evaluated in pull down assays (Figure 4E). To this end, DIIIE1–4 were used as bait and incubated with a microsomal fraction obtained from Huh7 cells, detecting bound LRP1 by Selected Reaction Monitoring in mass spectrometry. In this assay set up, both DIIIE2 and DIIIE4 were able to pull down the native LRP1 receptor as presented in the cell surface (Figure 4E and Table S1).

Altogether, these results demonstrate that the four serotypes of DENV bind the LRP1 receptor and that the DIII-mediated interaction is probably primary in binding to cluster IV of ligand-binding domains of this receptor. Even considering a possible influence of different assay formats, the DIII-mediated interaction is stronger with serotype 4, followed by serotype 2 and is weaker with serotypes 3 and 1.

Similar to other members of the family of the LDLR, LRP1 binding to its ligands has been proposed to occur through the acidic necklace model: amino groups of lysine residues of the ligands are encircled in a tripartite salt bridge via three remaining oxygen atoms from the acidic residues forming the octahedral calcium cage of LBDs [51]. It seems that there are modest requirements for the coordination of lysine residues [52], but specificity may be influenced by other residues located in its vicinity showing positive/negative charge [53,54] or hydrophobic character [55]. High-affinity binding seems to involve the interaction with two or more LBDs, requiring at least two lysine residues located at an appropriated distance—18–20 Å according to structural data –which allows the simultaneous binding of two consecutive LBDs [51]. Likewise, the relative contribution of a single lysine residue of a ligand protein to the interaction may be difficult to rationalize in terms of stereo-chemical properties, and the overall binding affinity may be the result of the additive effect of various weak binding sites [56].

The DIII contains 10–13 lysine residues depending on the serotype, but the number of basic residues is counted by the sum of aspartic and glutamic acids, resulting in the fact that, at neutral pH, the overall charge is predicted to be minimal for DENV1-DENV3 and only approximately +2 for DENV4 (Table S2). This observation highlights the relevance of specific interactions involving lysine residues of DIII, which interact with the receptor according to the acidic necklace model. The overall positive electrostatic potential of DIII from DENV4 may also contribute favorably to the interaction and explains in part why this serotype displays the higher binding affinity. Five lysine residues (K291, K295, K310, K334 and K394) are strictly conserved—in sequence—among the serotypes, but others are conserved topographically, i.e., the mutation of one lysine is accompanied by a compensating mutation to lysine in an adjacent position in the 3D structure, e.g., the mutation K388 (DENV1) to T (DENV4) is compensated by the mutation G344 (DENV1) to K (DENV4). Furthermore, there are several interatomic distances between the lysine residues of DIII, which are consistent with a putative interaction with two consecutive LBDs. Otherwise, the sequence identity among the DIII from the four serotypes varies between 52 and 71%, indicating that mutations close in sequence and/or 3D space to lysine residues would also affect the interaction and explain observed differences in binding affinity (Figure 2A and Table S3).

## 4. Discussion

Viral tropism is strongly determined, to a great extent, by the specific host–cell receptor(s) leveraged by a virus to infect a specific cell or tissue type [57]. Thus, the fact that DENV infects a dissimilar array of cell types including endothelial cells, fibroblasts, myeloid cells and lymphocytes [58] might be explained by the use of a single, ubiquitously expressed receptor or of several, varying cell receptors that are differentially expressed in distinct cell lineages.

A substantial number of different cell surface molecules have been shown to be involved in early stages of the DENV–cell interaction; most of these cases concern the first step of the cell entry process, i.e., adhesion [59]. The higher efficiency of infection afforded by the ectopic expression of DENV adhesion molecules, such as DC-SIGN, illustrates the importance of this initial interaction for the viral life cycle [60].

However, the following step, which, in the case of DENV, is cell entry by receptor-mediated endocytosis, is as important as the first. In the case of human cells, receptor-mediated endocytosis places the virion at the right cellular location and under the best conditions for membrane fusion [61]. Although TIM-1 is the only molecule that has convincingly been shown to internalize DENV by receptor-mediated endocytosis [20], both the experimental results of the characterization of TIM-1 activity during endocytosis [20,62] and the expression profile of this protein suggest that the virus must leverage other yet unknown molecules capable of receptor-mediated endocytosis.

For a cell receptor to be used as a viral port of entry, it must be able to bind the virus directly or through a bridging ligand. Our results show that LRP1 binds DENV virions (Figure 3C) and that this interaction competes, with potency at the nanomolar range, with DENV virion interactions involving other cell surface molecules (Figure 2E). Previous research has already shown that DENV virions can also bind LRP1 ligands [31,32,33], and in the case of α2M, it was proven that this interaction leads to increments in infection [33]. Whether DENV infection may proceed through the formation of a ternary DENV:α2M:LRP1 complex is the subject of ongoing research, but we would like to point out that the pathogenicity of WNV, a flavivirus closely related to DENV, is severely reduced in knockout mice defective for the expression of α2M homologs [63]. Lastly, it is worth noting that the assembly of ternary complexes is not exclusive to multivalent LRP1 ligands such as DENV and α2M but has been observed for other LRP1 ligands as well [64].

LRP1 is a highly efficient constitutive endocytic receptor mediating the internalization of over 40 different ligands by different mechanisms, including some human RNA viruses [65]. Minor group human rhinoviruses HRV2 were demonstrated to use LRP-1 and enter Hela cells via a clathrin-independent pathway, a mechanism that is significantly activated upon LRP1:HRV2 in the cell surface [66]. LRP-1 was also demonstrated to play an essential role for cell attachment and endocytosis of other viruses like Rift Valley Fever virus [67] and Oropouche virus in in vitro and experimental animal model assays [68]. Notably, similar to our results with DENV, for Rift Valley Fever virus and Oropouche virus, it was demonstrated that virus binding is mediated by extracellular regions LRP1-CII and LRP1-CIV [67,68]. Our results demonstrate that DENV efficiently binds LRP1. Also, this work provides evidence of a direct relation between the expression of LRP1 in human hepatocytes and the efficiency of DENV infection in Huh-7 cells (Figure 1). Together these two lines of results strongly suggest that LRP1 binding should result in a productive infection event. On the other hand, the fact that other viruses that constitute a concern to public health interact with LRP1 for infection makes this receptor an interesting target for the development of antiviral drugs with the potential to be effective in the treatment of various viral infections.

Nikolayeva et al. [69], identified LRP1 as one of the genes that exhibit lower cell surface expression in PBMCs of severe dengue patients. Taking into consideration that the isolation of PBMCs was performed from patients with a median of 3 days after the onset of the fever, DENV has replicated for at least 6 days at the moment that sample was taken for the study. The observed decrease in expression of the receptor is then not an event that can be associated with the first cycles of virus replication and dissemination in the human host but rather when consequences of infection have already risen. For other viral infections, it was described that, after initial cycles of infection, there is a decrease in the expression of cellular receptors, which is known as viral interference since it is a mechanism to prevent a subsequent infection [70]. Hence, this result associates LRP1 with the viral interference phenomenon [70], which, in light of our findings regarding the direct interaction with DENV virions, reinforces the notion that LRP1 is directly linked to the process of DENV infection in vivo and the pathogenesis of dengue disease. Whether LRP1 involvement in DENV infection is related to the virus entry stage or extends to the well-known participation of this molecule in diverse signaling and regulation processes with impact in virus post-entry events [30] remains to be determined.

LRP1 is well conserved evolutionarily [30]. Its *A. aegypti* ortholog (NCBI Reference Sequence: XP_021706493.1) exhibits a 35% sequence identity with the human protein, which is even higher (51%) across ligand-binding cluster IV, whose interaction with DENV is strongest (Figure 3C and Figure 4B). In contrast with our results of silencing LRP1 expression in Huh-7 cells, the silencing of LRP1 orthologs in mosquito cell lines Aag2 and c6/36 was reported to increase the copy number of viral RNA in infected cells [28]. In the same study, it was demonstrated that DENV infection triggers regulated intramembrane proteolysis of LRP1 leading to a significant decrease in the membrane-bound receptor [28]. Thus, considering that the modulation of cholesterol biosynthesis regulates DENV replication [71,72], the authors propose the possibility that DENV infection induces a decrease in LRP1 expression to avoid the control exerted by this receptor over intracellular cholesterol and the resulting impairment of viral replication [28]. Interestingly, regulated intramembrane proteolysis phenomenon begins upon ligand binding, which implies that there is an interaction between the DENV and LRP1 receptor at the cell membrane and is consistent with a similar role for this receptor in the early steps of infection in mosquitos—as in mammalian cells. In light of our present results, DENV inducing a decrease in LRP1 expression and at the plasma membrane could then be explained as a mechanism of negative viral interference to avoid cell superinfection [70]. In fact, recent results from our group are coherent with an important role of LRP1 promoting DENV infection in mosquitos. Our results show that a synthetic peptide, obtained by structure-based design to resemble the LRP1-binding surface in DIII of the E protein and block virus–receptor interaction [72], with demonstrated inhibitory activity against DENV1–4 infection in mammalian cells, exhibits antiviral activity against DENV-1 in mosquito cells in vitro in Aag2 and c6/36 and in vivo using a vector competence assay [manuscript submitted]. These latter results not only evidence an important role of the LRP1 receptor in DENV infection in mosquito cells but also positions this receptor as a prime target for the development of antiviral strategies.

One limitation of this study is that the effect of silencing LRP1 expression in Huh-7 cells was measured through virus yield in cell supernatant 24 h post-infection. Considering on one hand that the proposed role for LRP1 is an early event like virus entry to cells, and, on the other, that LRP1 is not only implicated in endocytosis but also in homeostasis of the intracellular milieu and signal transduction [73,74], a more direct measure of virus entry, like the detection of RNA of internalized virus, would had been more informative on whether this LRP1 has a prominent role in DENV entry to cells. Yet, jointly, our results strongly indicate that LRP1 plays a role in virus entry since a direct interaction of LRP1 located at the cell membrane with the virus was evidenced by means of a pull down experiment of recombinant DIII proteins with LRP1 present in microsomes of Huh-7 cells and the inhibition of infection by pre-incubation of the virus with the purified receptor. These results do not rule out the involvement of LRP1 in post-entry events though.

Our data prove that LRP1 binds DENV virions through protein E (Figure 3D,E) or, more specifically, through DIII of protein E (Figure 4), although they do not rule out the possible involvement of additional regions. DIII is the target of many highly potent neutralizing antibodies [75,76,77] that have been shown to disrupt and block early interaction events with cell surface molecules, presumably, the viral cell receptors [78]. This outcome, together with the immunoglobulin-like fold of this domain and the capacity of recombinant DIII to bind the cell surface [79] and compete with virus binding [6], has led many to consider it a receptor-interacting region, despite the fact that the only receptors DIII has been shown to bind are cell-surface glycosaminoglycans [7]. The results presented here indicate that the binding of protein E to LRP1 is conserved across all four DENV serotypes, although there are considerable inter-serotype differences regarding its affinity (Figure 4D).

It is also worth noting that several of the molecules described as DENV adhesion receptors are members of well-known LRP1 functional complexes. For instance, many LRP1 ligands use glycosaminoglycans as adhesion molecules [80], and proteins hsp90 [81], grp78 [82] and AXL [83], which were separately identified as DENV adhesion receptors [62,84,85], act cooperatively with LRP1 in binding, entry and signaling processes.

LRP1 is ubiquitously expressed, including several tissues and cell types identified as relevant for DENV replication such as monocytes/macrophages, keratinocytes, myeloid cells, phagocytes, hepatocytes and endothelial cells [30,86]. Thus, we hypothesize that LPR1 might constitute one of the hitherto unidentified endocytic receptors exploited by DENV to gain entry into mammalian cells.

## Data Availability

The raw data supporting the conclusions of this article will be made available by the authors upon request.

## Supplementary Materials

The following supporting information can be downloaded at: https://www.mdpi.com/article/10.3390/v16111692/s1, Table S1: Factor of enrichment of LRP1 proteotypic peptides with respect to the background control in the pull down experiments. The ^1^*p*-value calculated for the *t*-test analysis of each experimental condition means (DIIIE1, DIIIE2, DIIIE3 or DIIIE4) versus the background means of normalized intensity values. (*) denotes *p*-value below 0.05, (ns) no significance. The Skyline software ver. 21.1.0.146 was used to carry out the statistical analysis. Table S2: Isoelectric point and charge calculations. Table S3: Sequence identity* and similarity of DIII from DENV1–4. *values located over (under) the diagonal corresponds to sequence identity (similarity).

## References

[1] Guzman M.G., Gubler D.J., Izquierdo A., Martinez E., Halstead S.B. (2016). Dengue infection. Nat. Rev. Dis. Prim..

[2] Ilic I., Ilic M. (2024). Global Patterns of Trends in Incidence and Mortality of Dengue, 1990–2019: An Analysis Based on the Global Burden of Disease Study. Medicina.

[3] Hasan S.S., Sevvana M., Kuhn R.J., Rossmann M.G. (2018). Structural biology of Zika virus and other flaviviruses. Nat. Struct. Mol. Biol..

[4] Rey F.A. (2003). Dengue virus envelope glycoprotein structure: New insight into its interactions during viral entry. Proc. Natl. Acad. Sci. USA.

[5] Crill W.D., Roehrig J.T. (2001). Monoclonal Antibodies That Bind to Domain III of Dengue Virus E Glycoprotein Are the Most Efficient Blockers of Virus Adsorption to Vero Cells. J. Virol..

[6] Chin J., Chu J., Ng M. (2007). The envelope glycoprotein domain III of dengue virus serotypes 1 and 2 inhibit virus entry. Microbes Infect..

[7] Watterson D., Kobe B., Young P.R. (2012). Residues in domain III of the dengue virus envelope glycoprotein involved in cell-surface glycosaminoglycan binding. J. Gen. Virol..

[8] Chu J.J.H., Rajamanonmani R., Li J., Bhuvanakantham R., Lescar J., Ng M.-L. (2005). Inhibition of West Nile virus entry by using a recombinant domain III from the envelope glycoprotein. J. Gen. Virol..

[9] Zonetti L.F.D.C., Coutinho M.C., de Araujo A.S. (2018). Molecular Aspects of the Dengue Virus Infection Process: A Review. Protein Pept. Lett..

[10] Ayala-Nunez N.V., Hoornweg T.E., van de Pol D.P., Sjollema K.A., Flipse J., van der Schaar H.M., Smit J.M. (2016). How antibodies alter the cell entry pathway of dengue virus particles in macrophages. Sci. Rep..

[11] Dejnirattisai W., Supasa P., Wongwiwat W., Rouvinski A., Barba-Spaeth G., Duangchinda T., Sakuntabhai A., Cao-Lormeau V.-M., Malasit P., Rey F.A. (2016). Dengue virus sero-cross-reactivity drives antibody-dependent enhancement of infection with zika virus. Nat. Immunol..

[12] Shukla R., Ramasamy V., Shanmugam R.K., Ahuja R., Khanna N. (2020). Antibody-Dependent Enhancement: A Challenge for Developing a Safe Dengue Vaccine. Front. Cell Infect. Microbiol..

[13] Sarker A., Dhama N., Gupta R.D. (2023). Dengue virus neutralizing antibody: A review of targets, cross-reactivity, and antibody-dependent enhancement. Front. Immunol..

[14] Wells T.J., Esposito T., Henderson I.R., Labzin L.I. (2024). Mechanisms of antibody-dependent enhancement of infectious disease. Nat. Rev. Immunol..

[15] Li Y., Kakinami C., Li Q., Yang B., Li H. (2013). Human Apolipoprotein A-I Is Associated with Dengue Virus and Enhances Virus Infection through SR-BI. PLoS ONE.

[16] Chen Y., Maguire T., Hileman R.E., Fromm J.R., Esko J.D., Linhardt R.J., Marks R.M. (1997). Dengue virus infectivity depends on envelope protein binding to target cell heparan sulfate. Nat. Med..

[17] Jacobson K., Liu P., Ridilla M., Betts L., de Silva A., Thompson N.L. (2016). DC-SIGN Mediated Dengue Virus Entry into Cells. Biophys. J..

[18] Liu P., Ridilla M., Patel P., Betts L., Gallichotte E., Shahidi L., Thompson N.L., Jacobson K. (2017). Beyond attachment: Roles of DC-SIGN in dengue virus infection. Traffic.

[19] Anwar M.N., Akhtar R., Abid M., Khan S.A., Rehman Z.U., Tayyub M., Malik M.I., Shahzad M.K., Mubeen H., Qadir M.S. (2022). The interactions of flaviviruses with cellular receptors: Implications for virus entry. Virology.

[20] Dejarnac O., Hafirassou M.L., Chazal M., Versapuech M., Burlaud-Gaillard J., Perera-Lecoin M., Umana-Diaz C., Bonnet-Madin L., Carnec X., Tinevez J.-Y. (2018). TIM-1 Ubiquitination Mediates Dengue Virus Entry. Cell Rep..

[21] Lee C.-J., Lin H.-R., Liao C.-L., Lin Y.-L. (2008). Cholesterol Effectively Blocks Entry of Flavivirus. J. Virol..

[22] Biswas H.H., Gordon A., Nuñez A., Perez M.A., Balmaseda A., Harris E. (2015). Lower Low-Density Lipoprotein Cholesterol Levels Are Associated with Severe Dengue Outcome. PLoS Neglected Trop. Dis..

[23] Melo C.F.O.R., Delafiori J., Dabaja M.Z., de Oliveira D.N., Guerreiro T.M., Colombo T.E., Nogueira M.L., Proenca-Modena J.L., Catharino R.R. (2018). The role of lipids in the inception, maintenance and complications of dengue virus infection. Sci. Rep..

[24] Tree M.O., Londono-Renteria B., Troupin A., Clark K.M., Colpitts T.M., Conway M.J. (2019). Dengue virus reduces expression of low-density lipoprotein receptor-related protein 1 to facilitate replication in Aedes aegypti. Sci. Rep..

[25] Guevara J., Romo J., McWhorter T., Guevara N.V. (2015). Analogs of LDL Receptor Ligand Motifs in Dengue Envelope and Capsid Proteins as Potential Codes for Cell Entry. J. Viruses.

[26] Holtzman D.M., Herz J., Bu G. (2012). Apolipoprotein E and apolipoprotein E receptors: Normal biology and roles in Alzheimer disease. Cold Spring Harb. Perspect. Med..

[27] Chien Y.-J., Chen W.-J., Hsu W.-L., Chiou S.-S. (2008). Bovine lactoferrin inhibits Japanese encephalitis virus by binding to heparan sulfate and receptor for low density lipoprotein. Virology.

[28] Chen J.-M., Fan Y.-C., Lin J.-W., Chen Y.-Y., Hsu W.-L., Chiou S.-S. (2017). Bovine Lactoferrin Inhibits Dengue Virus Infectivity by Interacting with Heparan Sulfate, Low-Density Lipoprotein Receptor, and DC-SIGN. Int. J. Mol. Sci..

[29] Herz J. (2001). The LDL Receptor Gene Family. Neuron.

[30] Lillis A.P., Van Duyn L.B., Murphy-Ullrich J.E., Strickland D.K. (2008). LDL Receptor-Related Protein 1: Unique Tissue-Specific Functions Revealed by Selective Gene Knockout Studies. Physiol. Rev..

[31] Ramos Y., Huerta V., Martín D., Palomares S., Yero A., Pupo D., Gallien S., Martín A.M., Pérez-Riverol Y., Sarría M. (2019). An “on-matrix” digestion procedure for AP-MS experiments dissects the interplay between complex-conserved and serotype-specific reactivities in Dengue virus-human plasma interactome. J. Proteom..

[32] Huerta V., Ramos Y., Yero A., Pupo D., Martín D., Toledo P., Fleitas N., Gallien S., Martín A.M., Márquez G.J. (2016). Novel interactions of domain III from the envelope glycoprotein of dengue 2 virus with human plasma proteins. J. Proteom..

[33] Huerta V., Toledo P., Fleitas N., Martín A., Pupo D., Yero A., Sarría M., Sánchez A., Besada V., Ramos Y. (2014). Receptor-activated human α2-macroglobulin interacts with the envelope protein of dengue virus and protects virions from temperature-induced inactivation through multivalent binding. J. Gen. Virol..

[34] Huerta V., Ramos Y., Yero A., Pupo D., Martin D., Márquez G., Martín A., Sarría M., Gallien S., González L.J. (2016). Dataset on protein composition of a human plasma sub-proteome able to modulate the Dengue 2 virus infection in Huh 7.5 cells. Data Brief.

[35] Huerta V., Ramos Y., Mohana-Borges R. (2022). Isolation and Identification of Dengue Virus Interactome with Human Plasma Proteins by Affinity Purification-Mass Spectrometry. Dengue Virus.

[36] Laemmli U.K. (1970). Cleavage of Structural Proteins during the Assembly of the Head of Bacteriophage T4. Nature.

[37] Heukeshoven J., Dernick R. (1985). Simplified method for silver staining of proteins in polyacrylamide gels and the mechanism of silver staining. Electrophoresis.

[38] Towbin H., Staehelin T., Gordon J. (1979). Electrophoretic transfer of proteins from polyacrylamide gels to nitrocellulose sheets: Procedure and some applications. Proc. Natl. Acad. Sci. USA.

[39] De Winter J.C.F. (2013). Using the Student’s t-test with extremely small sample sizes. Pract. Assess. Res. Eval..

[40] Balsitis S.J., Coloma J., Castro G., Alava A., Flores D., McKerrow J.H., Beatty P.R., Harris E. (2009). Tropism of dengue virus in mice and humans defined by viral nonstructural protein 3-specific immunostaining. Am. J. Trop. Med. Hyg..

[41] Laatsch A., Panteli M., Sornsakrin M., Hoffzimmer B., Grewal T., Heeren J. (2012). Low Density Lipoprotein Receptor-Related Protein 1 Dependent Endosomal Trapping and Recycling of Apolipoprotein E. PLoS ONE.

[42] Zamule S.M., Strom S.C., Omiecinski C.J. (2008). Preservation of hepatic phenotype in lentiviral-transduced primary human hepatocytes. Chem. Biol. Interact..

[43] Finkelshtein D., Werman A., Novick D., Barak S., Rubinstein M. (2013). LDL receptor and its family members serve as the cellular receptors for vesicular stomatitis virus. Proc. Natl. Acad. Sci. USA.

[44] Strickland D.K., Ashcom J.D., Williams S., Burgess W.H., Migliorini M., Argraves W.S. (1990). Sequence identity between the alpha 2-macroglobulin receptor and low density lipoprotein receptor-related protein suggests that this molecule is a multifunctional receptor. J. Biol. Chem..

[45] Quinn K.A., Grimsley P.G., Dai Y.-P., Tapner M., Chesterman C.N., Owensby D.A. (1997). Soluble Low Density Lipoprotein Receptor-related Protein (LRP) Circulates in Human Plasma. J. Biol. Chem..

[46] Mukhopadhyay S., Kuhn R.J., Rossmann M.G. (2005). A structural perspective of the flavivirus life cycle. Nat. Rev. Microbiol..

[47] Thullier P., Demangel C., Bedouelle H., Mégret F., Jouan A., Deubel V., Mazié J.-C., Lafaye P. (2001). Mapping of a dengue virus neutralizing epitope critical for the infectivity of all serotypes: Insight into the neutralization mechanism. J. Gen. Virol..

[48] Hung J.-J., Hsieh M.-T., Young M.-J., Kao C.-L., King C.-C., Chang W. (2004). An external loop region of domain III of dengue virus type 2 envelope protein is involved in serotype-specific binding to mosquito but not mammalian cells. J. Virol..

[49] Larkin M.A., Blackshields G., Brown N.P., Chenna R., McGettigan P.A., McWilliam H., Valentin F., Wallace I.M., Wilm A., Lopez R. (2007). Clustal W and Clustal X version 2.0. Bioinformatics.

[50] Robert X., Gouet P. (2014). Deciphering key features in protein structures with the new ENDscript server. Nucleic Acids Res..

[51] Fisher C., Beglova N., Blacklow S.C. (2006). Structure of an LDLR-RAP complex reveals a general mode for ligand recognition by lipoprotein receptors. Mol. Cell.

[52] Jensen J.K., Dolmer K., Gettins P.G. (2009). Specificity of binding of the low density lipoprotein receptor-related protein to different conformational states of the clade E serpins plasminogen activator inhibitor-1 and proteinase nexin-1. J. Biol. Chem..

[53] Dolmer K., Campos A., Gettins P.G.W. (2013). Quantitative Dissection of the Binding Contributions of Ligand Lysines of the Receptor-associated Protein (RAP) to the Low Density Lipoprotein Receptor-related Protein (LRP1). J. Biol. Chem..

[54] Williams S., Inoue I., Tran H., Fry G., Pladet M., Iverius P., Lalouel J., Chappell D., Strickland D. (1994). The carboxyl-terminal domain of lipoprotein lipase binds to the low density lipoprotein receptor-related protein/alpha 2-macroglobulin receptor (LRP) and mediates binding of normal very low density lipoproteins to LRP. J. Biol. Chem..

[55] Jensen G.A., Andersen O.M., Bonvin A.M., Bjerrum-Bohr I., Etzerodt M., Thøgersen H.C., O’Shea C., Poulsen F.M., Kragelund B.B. (2006). Binding site structure of one LRP–RAP complex:Implications for a common ligand–receptor binding motif. J. Mol. Biol..

[56] Biggelaar M.v.D., Madsen J.J., Faber J.H., Zuurveld M.G., van der Zwaan C., Olsen O.H., Stennicke H.R., Mertens K., Meijer A.B. (2015). Factor VIII Interacts with the Endocytic Receptor Low-density Lipoprotein Receptor-related Protein 1 via an Extended Surface Comprising “Hot-Spot” Lysine Residues. J. Biol. Chem..

[57] Boulant S., Stanifer M., Lozach P.-Y. (2015). Dynamics of Virus-Receptor Interactions in Virus Binding, Signaling, and Endocytosis. Viruses.

[58] Noisakran S., Onlamoon N., Songprakhon P., Hsiao H.-M., Chokephaibulkit K., Perng G.C. (2010). Cells in Dengue Virus Infection In Vivo. Adv. Virol..

[59] Perera-Lecoin M., Meertens L., Carnec X., Amara A. (2013). Flavivirus Entry Receptors: An Update. Viruses.

[60] Navarro-Sanchez E., Altmeyer R., Amara A., Schwartz O., Fieschi F., Virelizier J., Arenzana-Seisdedos F., Desprès P. (2003). Dendritic-cell-specific ICAM3-grabbing non-integrin is essential for the productive infection of human dendritic cells by mosquito-cell-derived dengue viruses. EMBO Rep..

[61] van der Schaar H.M., Rust M.J., Chen C., van der Ende-Metselaar H., Wilschut J., Zhuang X., Smit J.M. (2008). Dissecting the Cell Entry Pathway of Dengue Virus by Single-Particle Tracking in Living Cells. PLoS Pathog..

[62] Meertens L., Carnec X., Lecoin M.P., Ramdasi R., Guivel-Benhassine F., Lew E., Lemke G., Schwartz O., Amara A. (2012). The TIM and TAM Families of Phosphatidylserine Receptors Mediate Dengue Virus Entry. Cell Host Microbe.

[63] Krause K., Azouz F., Nakano E., Nerurkar V.R., Kumar M. (2019). Deletion of Pregnancy Zone Protein and Murinoglobulin-1 Restricts the Pathogenesis of West Nile Virus Infection in Mice. Front. Microbiol..

[64] Pietrzik C.U., Jaeger S. (2008). Functional Role of Lipoprotein Receptors in Alzheimers Disease. Curr. Alzheimer Res..

[65] Devignot S., Sha T.W., Burkard T.R., Schmerer P., Hagelkruys A., Mirazimi A., Elling U., Penninger J.M., Weber F. (2023). Low-density lipoprotein receptor–related protein 1 (LRP1) as an auxiliary host factor for RNA viruses. Life Sci. Alliance.

[66] Bayer N., Schober D., Hüttinger M., Blaas D., Fuchs R. (2001). Inhibition of Clathrin-dependent Endocytosis Has Multiple Effects on Human Rhinovirus Serotype 2 Cell Entry. J. Biol. Chem..

[67] Ganaie S.S., Schwarz M.M., McMillen C.M., Price D.A., Feng A.X., Albe J.R., Wang W., Miersch S., Orvedahl A., Cole A.R. (2021). Lrp1 is a host entry factor for Rift Valley fever virus. Cell.

[68] Schwarz M.M., Price D.A., Ganaie S.S., Feng A., Mishra N., Hoehl R.M., Fatma F., Stubbs S.H., Whelan S.P.J., Cui X. (2022). Oropouche orthobunyavirus infection is mediated by the cellular host factor Lrp1. Proc. Natl. Acad. Sci. USA.

[69] Nikolayeva I., Bost P., Casademont I., Duong V., Koeth F., Prot M., Czerwinska U., Ly S., Bleakley K., Cantaert T. (2018). A Blood RNA Signature Detecting Severe Disease in Young Dengue Patients at Hospital Arrival. J. Infect. Dis..

[70] Piret J., Boivin G. (2022). Viral Interference between Respiratory Viruses. Emerg. Infect. Dis..

[71] Rothwell C., LeBreton A., Ng C.Y., Lim J.Y., Liu W., Vasudevan S., Labow M., Gu F., Gaither L.A. (2009). Cholesterol biosynthesis modulation regulates dengue viral replication. Virology.

[72] Osuna-Ramos J.F., Farfan-Morales C.N., Cordero-Rivera C.D., De Jesús-González L.A., Reyes-Ruiz J.M., Hurtado-Monzón A.M., Palacios-Rápalo S.N., Jiménez-Camacho R., Meraz-Ríos M.A., Del Ángel R.M. (2023). Cholesterol-Lowering Drugs as Potential Antivirals: A Repurposing Approach against Flavivirus Infections. Viruses.

[73] El Asmar Z., Terrand J., Jenty M., Host L., Mlih M., Zerr A., Justiniano H., Matz R.L., Boudier C., Scholler E. (2016). Convergent Signaling Pathways Controlled by LRP1 (Receptor-related Protein 1) Cytoplasmic and Extracellular Domains Limit Cellular Cholesterol Accumulation. J. Biol. Chem..

[74] Rauch J.N., Luna G., Guzman E., Audouard M., Challis C., Sibih Y.E., Leshuk C., Hernandez I., Wegmann S., Hyman B.T. (2020). LRP1 is a master regulator of tau uptake and spread. Nature.

[75] González-Lodeiro L.G., Dunn A.M., Prieto D.M., Medina-Carrasco D., de Castro L.E.G., Bauzá D.M., Santiago G.C., Galindo V.H. (2024). Dominant epitopes of cross-reactive anti-domain III human antibody response change from early to late convalescence of infection with dengue virus. J. Med. Virol..

[76] Medina-Carrasco D., Pupo D., González-Lodeiro L.G., García L.E., Martin A.M., Huerta V. (2023). Activity of domain III-specific antibodies in early convalescence: A case study. Virology.

[77] Zhou Y., Chen D., Yang L., Zou W., Duan Z., Zhang Y., Wen J. (2020). Dengue virus envelope protein domain III-elicited antibodies mediate cross-protection against Zika virus in a mouse model. Virus Res..

[78] Fahimi H., Mohammadipour M., Kashani H.H., Parvini F., Sadeghizadeh M. (2018). Dengue viruses and promising envelope protein domain III-based vaccines. Appl. Microbiol. Biotechnol..

[79] Saotome T., Doret M., Kulkarni M., Yang Y.-S., Barthe P., Kuroda Y., Roumestand C. (2019). Folding of the Ig-Like Domain of the Dengue Virus Envelope Protein Analyzed by High-Hydrostatic-Pressure NMR at a Residue-Level Resolution. Biomolecules.

[80] Kanekiyo T., Zhang J., Liu Q., Liu C.-C., Zhang L., Bu G. (2011). Heparan Sulphate Proteoglycan and the Low-Density Lipoprotein Receptor-Related Protein 1 Constitute Major Pathways for Neuronal Amyloid-β Uptake. J. Neurosci..

[81] Gopal U., Bohonowych J.E., Lema-Tome C., Liu A., Garrett-Mayer E., Wang B., Isaacs J.S. (2011). A Novel Extracellular Hsp90 Mediated Co-Receptor Function for LRP1 Regulates EphA2 Dependent Glioblastoma Cell Invasion. PLoS ONE.

[82] Misra U.K., Mario G.G., Gawdi G., Wang F., Pizzo S.V. (2004). A novel receptor function for the heat shock protein Grp78: Silencing of Grp78 gene expression attenuates α2M-induced signaling. Cell. Signal..

[83] Subramanian M., Hayes C.D., Thome J.J., Thorp E., Matsushima G.K., Herz J., Farber D.L., Liu K., Lakshmana M., Tabas I. (2014). An AXL/LRP-1/RANBP9 complex mediates DC efferocytosis and antigen cross-presentation in vivo. J. Clin. Investig..

[84] Jindadamrongwech S., Thepparit C., Smith D.R. (2004). Identification of GRP 78 (BiP) as a liver cell expressed receptor element for dengue virus serotype 2. Arch. Virol..

[85] Reyes-del Valle J., Chávez-Salinas S., Medina F., del Angel R.M. (2005). Heat Shock Protein 90 and Heat Shock Protein 70 Are Components of Dengue Virus Receptor Complex in Human Cells. J. Virol..

[86] Pallesen G., Moestrup S.K., Gliemann J. (1992). Distribution of the *α*_2_-macroglobulin receptor/low density lipoprotein receptor-related protein in human tissues. Cell Tissue Res..

