# Supplementary material for: The Low-Density Lipoprotein Receptor-Related Protein-1 Is Essential for Dengue Virus Infection
Source: Viruses. 2024 Oct 30;16(11):1692. doi: 10.3390/v16111692 (PMC11599027; doi:10.3390/v16111692)
Supplement: Supplementary file 1 [file viruses-16-01692-s001.zip › viruses-3237755-supplementary.pdf]

Supplementary Materials

The low-density lipoprotein receptor-related protein-1 is essential for Dengue virus infection

Vivian Huerta[1*], Alejandro M. Martin[1], Mónica Sarría[1], Osmany Guirola[1], Alexis Yero[1#a], Yassel Ramos[1], Dianne Pupo[1#b], Dayron Martin[1#c], Luis G. González-Lodeiro[1], Alessandro Marcello[2] and Glay Chinea[1]

[1]. Department of System Biology, Direction of Biomedical Research, Center for Genetic Engineering and Biotechnology, Havana, Cuba

[2]. Laboratory of Molecular Virology, International Centre for Genetic Engineering and Biotechnology, Trieste, Italy; marcello@icgeb.org

[#a]Current Address: Center for Immunology and Inflammatory Diseases, Massachusetts General Hospital, Boston, MA. Harvard Medical School, Boston, MA. Division of Rheumatology, Allergy, and Immunology, Massachusetts General Hospital, Boston, MA, United States. Email: ayerodiaz@mgh.harvard.edu

[#b]Current Address: Department of Immunology and Cell Biology. Université de Sherbrooke, Québec, Canada. Email: dianne.pupo.gomez@usherbrooke.ca.

[#c]Current Address: Sericulture group, Experimental station of pastures and forages Indio Hatuey, Cuba. Email: dayron.martin@ihatuey.cu

[*]Corresponding author: vivian.huerta@cigb.edu.cu (VH)

Table S1

Factor of enrichment of LRP1 proteotypic peptides respect to the background control in the Pull Down experiments.

Peptides	Factor of Enrichment [P-value][1]			
	DIIIE1	DIIIE2	DIIIE3	DIIIE4
^{365}IVFPHGITLDLVSR.L^{378}	1.49^{ns}	4.61*	1.55^{ns}	8.61*
^{696}TVLWPNGLSLDIPAGR^{711}	1.13^{ns}	3.51*	2.89*	7.62*
^{1814}ADGSGSVVLR^{1823}	1.33^{ns}	2.35*	1.05^{ns}	3.47*
^{2360}AALSGANVLTLIEK^{2373}	1.48^{ns}	5.69*	1.67^{ns}	7.73*
^{3022}AVTDEEPFLIFANR^{3035}	1.04^{ns}	2.13*	1.12^{ns}	5.13*
^{3861}AEGSEYQVLYIADDNEIR^{3878}	1.48^{ns}	2.22*	1.05^{ns}	5.21*

[1] P-value calculated for the T-test analysis of each experimental condition means (DIIIE1, DIIIE2, DIIIE3 or DIIIE4) versus the background means of normalized intensity values. (*), denotes P-value below 0.05, (ns) no significative. The Skyline software was used to carry out the statistical analysis.

Table S2

Isoelectric point and charge calculations

Serotype Sequences	Number of residues					Max Net charge	Expasy* pI	Protein Calculator Putnam**				IPC protein Kozlowski***		
								pI	charge			pI	charge	
	K	R	H	E	D				pH 5.5	pH 7	pH 7.4		pH 5.5	pH 7.4
Domain III														
DV1	13	1	1	10	4	1	6.58	7.09	1.9	0.1	-0.3	6.51	1.4	-1.1
DV2	11	3	2	9	5	2	6.72	7.28	2.8	0.3	-0.1	6.62	1.9	-1
DV3	13	1	2	11	4	1	6.11	6.53	1.9	-0.6	-1.1	5.92	1.1	-2.1
DV4	10	4	2	9	3	4	8.58	8.45	4.7	2.3	1.9	7.87	3.9	1
Rec-protein														
DIIIE1	13	1	7	11	4	6	6.65	7.19	6.5	0.6	-0.6	6.4	4	-2
DIIIE2	11	3	8	10	5	7	6.70	7.23	7.4	0.8	-0.5	6.45	4.5	-1.9
DIIIE3	13	1	8	12	4	6	6.43	6.95	6.4	-0.2	-1.5	6.16	3.5	-3
DIIIE4	10	4	8	10	3	9	7.86	8.08	9.2	2.8	1.5	7.44	6.4	0.1

Table S3

Sequence identity* and similarity of DIII from DENV1-4

	DV1	DV2	DV3	DV4
DV1	100	67.0	71.4	56.2
DV2	80.4	100	61.6	60.7
DV3	88.4	81.2	100	51.8
DV4	72.3	79.5	71.4	100

*values located over (under) the diagonal correspond to sequence identity (similarity)
